# *“You just can’t do that in dementia care”:* Barriers to partnership working within dementia services for people from south Asian communities

**DOI:** 10.1177/14713012241283189

**Published:** 2024-09-14

**Authors:** R Cheston, E Dodd, P Smith, NS Woodstoke, K Jutlla, G Fry, D Truswell, J Butt, S Parveen

**Affiliations:** School of Social Sciences, 1981 University of the West of England, UK; School of Health and Social Wellbeing, 1981 University of the West of England, UK; Department of Psychology, 1555 University of Bath, UK; School of Social Sciences, 1981 University of the West of England, UK; Faculty of Education, Health and Wellbeing, 8695 University of Wolverhampton, UK; Centre for Applied Dementia Studies, Faculty of Health, 1905 University of Bradford, UK; The Dementia Alliance for Culture and Ethnicity, UK; The Race Equality Foundation, UK; Centre for Applied Dementia Studies, Faculty of Health, 1905 University of Bradford, UK

**Keywords:** minority health, Alzheimer’s disease, voluntary health agencies, caregivers, accessibility of health services, community health care

## Abstract

**Background:** People from South Asian communities are under-represented at all levels of dementia services. Consequently, there is pressure for the statutory sector to deliver services in partnership with Voluntary, Community, Faith and Social Enterprises (VCFSEs). This study set out to explore the constraints to effective partnership working which prevent dementia care from being delivered in an equitable way.

**Methods:** Data collection consisted of two phases. First, we interviewed seven people with experience of partnership working and developed three fictional vignettes that were representative of the challenges they faced. We then used these vignettes to stimulate discussion in focus groups and interviews with 13 VCFSE and 16 statutory sector participants. Data was analysed using deductive thematic analysis.

**Findings:** Three themes were developed during the analysis. First, White British-centric services focused on the challenges for statutory services in meeting the needs of South Asians, developing flexible, responsive services and making inclusive partnership working truly meaningful. Second, VCFSE participants (but not statutory service participants) associated a failure to deliver effective partnership working with unconscious bias operating within systems, leading to the devaluing of their expertise and to their views being ignored. Finally, participants emphasised the need to prioritise relationships if they were to meet the challenges of developing partnership working.

**Conclusion:** We identified three constraints acting to prevent effective partnership working. First, the different meanings that statutory and VCFSE participants attach to challenges threatens their ability to develop a shared understanding of the needs of communities. Second, a reluctance to explicitly address service deficiencies can mean that stereotypes remain unaddressed. Finally, while both parties lacked power to change the fundamentals of service delivery, power and resources were also unbalanced with VCSFE services being more reliant on the statutory sector.

## Introduction

People from South Asian communities make up the second largest ethnic community living in the UK, with just over four million people, representing roughly seven per cent of the population in England and Wales, identifying themselves as Asian/Asian British Indian, Pakistani or Bangladeshi according to the 2021 census^1^. South Asian communities include Sri Lankans, as well as third-generation Asians, Asians of mixed parentage, people from Nepal, Bhutan and the Maldive Islands and some people from the Middle East (Jutlla & Arblaster, 2023). Compared to the White British population, South Asian communities are more at risk of being affected by dementia (Mukadam et al., 2022) with the number of people living with dementia expected to increase seven-fold by 2051, compared to a doubling in overall numbers across the UK (Wohland et al., 2010).

Dementia care for people from South Asian communities in the UK is marked by a series of health inequalities (Shafiq et al., 2024). Diagnosis is more likely to occur at a later stage when the patient is often more severely impaired or in crisis (Hailstone et al., 2017). Consequently, South Asians are less likely to receive NICE-recommended treatments, including medication for Alzheimer’s disease (Baghirathan et al., 2020; Parveen et al., 2017). People from South Asian communities who are living with dementia are also less likely to access the dementia care pathway (APPG, 2013) and when they do are more likely to evaluate NHS dementia services negatively (Mukadam et al., 2011). Many older South Asians rely on support from community groups whose staff and volunteers are not dementia trained (Jutlla & Arblaster, 2023) partly as a consequence of their poorer fluency in spoken or written English (Blakemore et al., 2018; Hussain et al., 2024). South Asians with dementia are more likely to be cared for at home and are less likely to have access to specialist care and support (Dodd et al., 2022; Duran-Kiraç, Uysal-Bozkir, Uittenbroek, van Hout, & Broese van Groenou, 2022). They tend to have a poorer quality of life and to be lonely whilst carers experience higher levels of stress and role captivity whilst having a lower relationship quality than do their White British peers (Victor et al., 2023). South Asians who are living with dementia tend to be under-represented in dementia services (Blakey et al., 2016) and are socially and financially disadvantaged compared to their White British counterparts (James et al., 2023).

A series of initiatives have attempted to address these health inequalities. This includes education campaigns to improve levels of awareness about dementia (Uppal & Bonas, 2014) so that symptoms of dementia are recognised as an illness (Werner et al., 2014) rather than as normal ageing (Bhattacharyya & Benbow, 2013; Brooker et al., 2014). Increasingly dementia services are turning to partnerships with locally rooted Voluntary, Community, Faith and Social Enterprises (VCFSEs) (Blakey et al., 2016) in order to compensate for service deficits including a lack of skilled interpreters and poor connections with the local community connections (Bhattacharyya & Benbow, 2013; Farooq et al., 2015; Parveen et al., 2017).

### Partnership working between statutory and VCFSE sector organisations

Partnership working between health and social care are an important prerequisite of strategies to tackle health inequalities (Evans & Killoran, 2000) and have been a central feature of policy directives not just in the UK but across Europe and North America (Petch et al., 2013). Partnership working spans a continuum from tentative collaboration between individuals to joint commissioning, strategic alliances and even full integration through a combination into a single agency (Petch et al., 2013). The identified benefits of partnership working include more coherent and effective service delivery, enhanced understanding of differing roles and the creation of a shared culture across services (Petch et al., 2013). A range of principles have been identified as underpinning effective partnership working including trust, equality, respect and mutual accountability (Hatton & Schroeder, 2007).

Whilst cross-sector partnerships working can bring benefits they face different challenges, however, which may compromise their effectiveness. These can include inequalities in power between larger, national organisations, and smaller, community-specific groups (Blakey et al., 2016; Minkler, 2005), the inadequate sharing of resources (Dowling et al., 2004; Petch et al., 2013; Taylor-Robinson et al., 2012) and a failure to create equity of participation and accountability (Boydell & Rugkåsa, 2007; Dickinson & Glasby, 2010; Evans & Killoran, 2000). These challenges are especially likely to impact on VCFSEs which typically play a crucial role in both giving voice to their communities’ needs whilst also pressing for greater choice and access to service provision (Blakey et al., 2016).

Given the range of potential hazards that partnership working can face, a number of different models of successful working have been put forward (Hatton & Schroeder, 2007). One such model (Boydell & Rugkåsa, 2007) was developed from Pawson and Tilley’s (1997) realist evaluation framework that suggests that successful outcomes are the product of an interaction between the mechanisms involved within a complex intervention and the wider context that it operates within. As mechanisms have different effects according to the context within which they operate, in developing their model Boydell and Rugkåsa focused on understanding what works for whom in what conditions (Pawson & Tilley, 1997) within two health action zones in Northern Ireland. Their model holds that successful partnership working relies on four linked elements. As partnership forms, partners connect with one another and develop relationships that allow each of them to begin working with networks outside the partnership. By starting to get to know each other, so partners learn about each other’s organizations, priorities and work and develop a more holistic understanding of local communities’ needs. This enables them to begin to develop trust in each other. As a result of this knowledge, understanding and trust, partners find that this helps them to do their jobs better. Finally, these developments can lead to more effective projects, improved service delivery and strengthening of communities.

At the same time, partnership working is likely to face both internal and external constraints, which need to be resolved as this process of working develops. Internal constraints are those factors that are inherent to the partnership itself, such as conflict between partners and the time-consuming nature of collaborative work. External constraints on the other hand, are those things which are beyond the control of the partnership, but which impact on its ability to achieve its goals. These may include changing and conflicting policies, the availability of resources and the wider political climate which it operates within (Boydell & Rugkåsa, 2007).

### Research question

Much of the research into partnership working has focused on the challenges that can arise at an organisational or senior leadership level (e.g., Alzheimer’s Society, 2018a, 2018b; Charles et al., 2018; Ham & Walsh, 2013). By contrast the experiences of practitioners working across services has been comparatively neglected (Chase et al., 2021). This perspective may be especially important for dementia services for South Asians which have to negotiate not just organisational but often language and cultural barriers as well. This study therefore aims to explore the internal and external constraints around partnership working which workers in the statutory sector and VCFSEs face as they attempt to work together to deliver equitable dementia care to people from South Asian communities.

## Method

To create a focal point for discussion about the challenges of partnership working, we created three fictional but illustrative accounts of potential challenges to partnership working. We then asked two separate populations (statutory sector and VCFSE staff) to respond to these vignettes. We registered our protocol^2^ and received ethical approval^3^ before commencing data collection. We followed the COREQ guidance for reporting qualitative research (Tong et al., 2007).

### Phase one: Vignette development

To develop the three vignettes, we interviewed seven participants online following a semi-structured interview topic guide based on the themes generated from our previous work (Blakey et al., 2016). We purposively recruited participants with experience of working at different levels of social and health care either within Black and Minority Ethnic led VCFSE sector organisations or NHS provided services (see Table 1).

**Table 1.** Details of participants interviewed to generate data for phase one (vignette development).

ID	Sex	Ethnicity	Occupation, experience of partnership working
1	Female	White-British	Over 10 years of working in the voluntary sector
2	Male	African-Caribbean	Worked in dementia care for an NHS trust, and in equalities and diversity role for a national organisation
3	Female	African-Caribbean	Dementia worker with over 10 years’ experience in different roles in statutory and voluntary sectors
4	Female	South Asian	Experience in a senior leadership position for a south Asian community organisation for over 15 years
5	Female	White-British	Dementia care trainer for social care staff, and experience as a manager of a day centre within the voluntary sector
6	Female	White-British	Has more than ten years nursing and social work experience within dementia care
7	Female	White-British	Community engagement officer for NHS trust specialising in dementia care

Interviews lasted up to 90 minutes and were conducted online by ED and PS (both identifying as female) and RC (who identifies as male), all of whom are white, have doctoral level qualifications in social science subjects and are experienced qualitative researchers. Interviews were audio-recorded and then transcribed by the same research team. To ensure good construct validity, we followed the recommendations of Evans et al. (2015) for generating vignettes. The initial drafts of vignettes were shared with SP and two public contributors (all South Asian females) who recommended changes, including making clearer the role of unconscious bias. Each of the three vignettes (see Appendix One) described a different challenge faced by Sunita (a fictional character), who “*manages a small community organisation providing support to older people from south Asian communities, including people who may be living with dementia*”. The vignettes related to three different challenges within partnership working: acknowledging service inequality in commissioning (vignette one), acknowledging expertise in joint working (vignette two), and sharing expertise across organisations (vignette three). The vignettes were each followed by a series of prompts intended to trigger discussion.

### Phase two: Recruitment and data collection

We then used these three vignettes to facilitate discussions around partnership working within focus groups recruited from two sources: staff and volunteers working for VCFSE sector organisations; and NHS and adult social care staff. Both groups had experience of providing services to people from South Asian communities living with dementia. The two groups of stakeholders were kept separate to create a safe space for open discussion about challenges of partnership working. Advertising for participants was done through posters, email and social media. Potential participants were invited to book into a focus group via an online platform, where they were also asked to provide brief demographic details (see Tables 2 and 3). Wherever possible we arranged for participants to take part in online focus groups in order to enable discussion of the vignettes, but in three cases (all with statutory sector participants) this was not possible and instead we collected data through one-to-one interviews. Data collection (in either focus groups or interviews) lasted up to 90 minutes and were conducted on Microsoft Teams by ED, PS and RC. Three participants (Anya, Kayleigh and Mitchell) were previously known to ED and RC. Verbal consent was obtained, and sessions were recorded. We have used pseudonyms throughout to refer to participants.

**Table 2.** Participant details for focus groups run with workers with Voluntary and Community organisations.

Focus group	Name^a^	Age	Self-described ethnicity	Sex	Role
A	Sophie	42	White	Female	Dementia community worker for a national VCSO
	Sana	25	Pakistani	Female	Service manager for local dementia VCSO
	Babar	45	Pakistani	Male	Dementia community worker for local branch of a national VCSO
	Nida	38	Pakistani	Female	Dementia community worker for dementia VCSO
	Javeria	63	Pakistani	Female	Dementia community worker for local dementia VCSO
	Mithali	-	British Indian	Female	Services manager for local branch of a national VCSO
B	Laura	56	White British	Female	Service manager for health care support group
	Sarah	29	White British	Female	Service manager for health care support group
	Anam	21	Bangladeshi	Female	Dementia community worker for local dementia VCSO
	Fakhar	67	Pakistani	Male	Dementia community worker for local branch of a
	Rehan	-	British Pakistani	Male	national VCSO
	Jasprit	-	British Indian	Male	Independent consultant
	Imad	-	British Pakistani	Male	Service manager for local branch of a national VCSO Community worker for local branch of a national VCSO

^a^The names of all participants have been altered.

**Table 3.** Participant details for interviews with workers for NHS and Council organisations.

Focus group/1-2–1 interview	Name^*^	Age	Self-described ethnicity	Sex	Role
C	Anya	-	White-British	Female	Service manager, NHS trust
D	Danni	43	White British	Female	Adult social care manager, city council
	Alice	54	White British	Female	Community psychiatric nurse, NHS trust
	Kate	47	White British	Female	Clinical psychologist/clinical lead, NHS trust
E	Nat	-	White British	Female	Clinical psychologist, NHS trust
F	Mitchell	56	White British	Male	Consultant psychiatrist, NHS trust
G	Amy	53	White British	Female	Community psychiatric nurse, NHS trust
	Issy	34	White British	Female	Social care practitioner, council
	Emma	58	Black Caribbean	Female	Community psychiatric nurse, NHS trust
	Tasmin	38	White British	Female	Social worker, council/NHS trust
H	Lydia	49	White British	Female	Clinical psychologist, NHS trust
	Lauren	26	Chinese	Female	Research assistant, NHS trust
	Jenny	34	White British	Female	Clinical psychologist, NHS trust
I	Jade	54	White Irish	Female	Service manager, NHS trust
	Leah	37	White British	Female	Community psychiatric nurse, NHS trust
	Kayleigh	63	White British	Female	Manager and inclusions officer for CCG

* The names of all participants have been altered.

### Data analysis

During the analysis, a fourth researcher, NW, was added to the analytical group of ED, PS and RC. NW identifies as female and is a white researcher, with a doctoral level qualification in clinical psychology. The analytical team shared the transcribing of the recorded focus groups and interviews and then used deductive thematic analysis to analyse the transcripts. Deductive thematic analysis is an established and well used form of qualitative analysis (e.g., Lambert & O'Halloran, 2008; Merriman et al., 2021; Sinclair-Maragh & Bernard Simpson, 2021) and has been well described in the methodological literature (e.g., Braun & Clarke, 2012; Terry et al., 2017). This approach to analysis is especially relevant when, as in this case, the research objectives are clear, and the theoretical framework is well-established. We based the initial shaping of the data in terms of a series of preconceived codes (detailed in Appendix two) derived from the relevant literature (Boyatzis, 1998; Braun & Clarke, 2012; Pearse, 2019). Working from this departure point, the analytical team then systematically worked through each transcript to code data relevant to each of these codes, noting where the revision of an existing code was necessary or where material could not be incorporated into the framework and a new code was required. These were then discussed in two meetings where preliminary themes drawing together these codes and drafting relationships between them were mapped out to give a visual idea of the relationships and patterns that the team was identifying in the data. PS then made an initial draft analysis bringing together and reviewing the map of the preliminary themes against the collated data at an extract level and across the entire dataset. This was then shared with ED, RC and NW and further iterations of coding, reordering and regrouping occurred until the themes and subthemes were judged to reflect the key meanings in the data and to demonstrate both internal homogeneity and external heterogeneity (Patton, 1990). During this process, naming and defining the themes and sub-themes took place against the context of identifying the core narratives and extracts needed to summarise them. The main themes were then shared with SP, KJ and the two public contributors to the project (all South Asian women) either in an online meeting or via email.

## Findings

We identified three themes.

### Theme 1: White British-centric services

In all but one of the focus groups and interviews, participants described how statutory services lacked the skills and knowledge necessary to deliver care equitably – at least partly because the workforce was predominantly white. As Nat, a white NHS clinical psychologist, told us: “*You need a more ethnically diverse workforce really, clinically, across th*e board”. This created challenges for services such as delivering person-centred in the language that people using the service spoke – whether that was Punjabi, Gujrati, Bengali, Hindi or Urdu. As Mitchell commented: “*how can you provide any kind of service if you can't speak the primary language … you just can’t do that in dementia care*”.

#### “It’s like well you have to fit into our service”: Flexible and responsive services

A number of VCFSE participants described how many health care professionals knew little about Islamic, Hindu or Sikh religious practices, such as the imperative for Muslims of observing a daylight fast during Ramadan, and were therefore at risk of providing services that many clients would feel demeaned them:

I was really shocked that I was speaking to professionals, and they did not know the month of Muharram … which is the first [month] in the Islamic year ... And a lot of people follow the Shia during that month (Nida).

For Imad, delivering a person-centred service meant that he needed to take a flexible approach to accommodate the cultural and religious needs of his clients. This meant leaving behind a “*nine to five*” mentality so that he could go “*to talks at two o’clock in the morning in June, Ramadan after prayer”*. However, whilst both VCFSE and statutory sector participants identified the importance of more flexible services, they tended to identify different ways of fitting services to communities. Participants working for statutory services tended to focus on changing individual practice. Thus, for example Alice discussed how she moved away from formal health settings to better engage the community in settings where they felt more comfortable: “*We didn’t use mental health buildings at all … doing talks in mosques, at community events I’d go and do a stand, talk to people about what memory was*”. In contrast, for VCFSE participants, the key to building flexible and responsive services was to fundamentally redesign how those services were delivered based around a better understanding of the needs of their community:

Whatever model that they've got … is actually designed for the majority of the service people that use (it), which are generally white people (Mithali).

It's got to come from the bottom up, not from top down and I think that's where we get confused, and we think that this is what our communities need now. Let's listen to those communities …. what makes them click (Babar).

Similarly, Nida (a community worker in the voluntary sector) claimed that commissioners *“don't understand what they’re trying to create, what service or what they are commissioning for … what are the communities living in the area, what services they need”*. For other VCFSE participants, engaging service commissioners wasn’t just hampered by a lack of knowledge, but by a lack of empathy. For instance, one participant described how he had been invited by a national charity to attend a planning meeting in London:

And they talked about, you know, points of reference, what can you talk about when someone may regress back in time. And in their wisdom, they thought 9/11 would be a really good subject for them to talk about in the South Asian community, and … I'm looking up in “am I the only one?” (Babar).

#### “Of course, it’s not ideal”: Prioritising the problem

Several of the statutory sector participants had worked in senior positions within their services, for instance as clinical leads (Kate, Mitchell), or service managers in the NHS (Anya, Jade and Kayleigh) or adult social care (Danni). These participants responded to the vignettes in part by identifying their own experiences of providing care services that had not been able to meet the needs of South Asian clients, and the steps they had taken to address this, for instance by seeking out partnerships with local, South Asian led VCFSEs. As Anya commented:

I think we have to just be, be honest about it that it is an issue an, and huh, it is regrettable … when people who have dementia, someone that is not speaking your language is clearly not going to be a gold star care is it? … but if we haven’t got those resources and there is absolutely no chance that we're going to get those resources, which realistically there isn’t, then we have to be just open and honest about it (Anya).

While Anya’s open recognition of a service shortfall is an important first step in recognising the need for change, nevertheless this can only happen if the service prioritises the need to resolve the problem. However, one possible reading of Anya’s framing of care as “a gold star” is that this positions care being delivered in the language that people speak as an ideal to be aimed at rather than as a basic element of person-centred care. This interpretation of Anya’s account is supported by Mitchell’s somewhat cynical account in a separate focus group of a tendency within the NHS to define challenging service delivery problems out of existence if they are deemed too difficult to resolve:

Right, I love that, going back to find paid carers who speak Punjabi or Urdu, they don't see how this can be solved and feel on balance it doesn’t detract from the quality of care that's provided. That's a classic NHS answer. We can't solve it, so it's not a problem. So, we won't commission anything that could solve the problem. We're trying to determine that it’s actually not a problem (Mitchell).

While there was widespread agreement then that many statutory services struggle to deliver appropriate care, both Anya and Mitchell, albeit in different ways appear to cast some doubt on the commitment within services to prioritise the changes that are needed.

#### “We’re only there to tick a box”: Is inclusivity meaningful?

To overcome the deficiencies in their services, some statutory services turned to local, South Asian led community organisations to provide the expertise that they lacked. However, unless there was a genuine commitment to partnership working, then any greater involvement of VCFSEs risked being seen as a performative gesture. Thus, both VCFSE and statutory sector participants could be cynical about the motives of managers, referring to a ‘tick-box’ mentality in which the importance of greater inclusivity was addressed in name only. As one VCFSE participant commented:

A lot of meetings, I know I’ve attended many of them, we're only there to tick a box we have contact with one of the BME … In real terms it means nothing (Jasprit).

Similarly, some statutory service healthcare workers articulated their frustrations at being constrained within a model of service delivery which Kate described as being *“all about targets, in performance indicators?”*. In this context she felt identifying the needs of South Asian clients could give rise to a conflict with managers between “what you want to do versus what you're allowed to do, what you’re commissioned to do” and which led on occasion to people *“feeling a little bit used, that sometimes* *they might be helping people to meet their goals, and you know tick their boxes … it needs to be a reciprocal relationship for it to be successful*” (Kate). Within this framing partnership work was represented as more about enhancing their trust’s reputation than reflecting a genuine commitment to inclusive practice. As Mitchell argued, *“It's something to put in the annual trust report … and to contact the local paper, say that we're really good at this”.*

## Theme 2: Unconscious bias or structural racism?

In responding to our vignettes, participants faced an implicit dilemma: whether or not to attribute the challenges that Sunita faced to at best unconscious bias, and at worst a form of structural racism. Participants were divided as to whether the challenges depicted in the vignettes were true either to their working lives or their lives more generally. While we did not explicitly ask participants about this, nevertheless it was noticeable that where the relevance of the vignettes was questioned then this was within the focus groups with statutory sector participants. Thus, Nat was typical of some participants from the statutory sector who did not recognise vignette two as being representative of the services that she worked in, saying: “*I can’t imagine that happening where I am. Locally I hope it would never happen*”. In contrast, Mithali’s response to the same vignette was typical of many VCFSE participants when he described it as being “*very real, that vignette*”. For many VCFSE participants, the challenges their clients faced in accessing appropriate care mirrored not just their working lives but extended to their own day-to-day experiences of struggling against unconscious bias within systems. Thus, within these accounts the deficiencies of the dementia care system were located within more general inequalities and social prejudices:

We as BAME communities feel and we have to try and justify ourselves … than what a white colleague might have to, and we have to try ten times harder … Other people need to start understanding what BAME communities gone through for such a long time (Imad).

### “Expertise without acknowledgement”: Valuing work appropriately. 

VCFSE participants described South Asians as being more likely to disengage from services when they felt that the dementia care that their relatives would encounter was likely to be indifferent to their needs. In this sense, refusing outside help and providing care themselves was perhaps one of the only ways that South Asians could be confident that this care did not compromise their relatives’ identity:

We might have this bias of well you’re OK because you look after your own – well do we look after our own? or is it the fact that we are petrified that I as a Muslim person or someone that is of a Hindu background or a Sikh background is fearful that if my mum, my brown mother goes into care in an institution she might be given something that she would never eat knowingly … [such as] a ham sandwich? who’s to say that won’t happen? (Rehan).

VCFSE participants often linked the service shortfalls that their clients faced to the wider disparity between the status of the organisations they worked for and those of the statutory sector: while their own funding was tied to short-term contracts and thus inherently unstable, their counterparts within the NHS were salaried staff on permanent contracts. However, NHS workers typically lacked the expertise to engage with South Asian communities and so relied on VCFSE staff and volunteers to make up for these service deficiencies. Often this took place without truly acknowledging the power imbalances. Thus, Rehan responded to vignette two where two NHS clinicians worked with Sunita to deliver a group:

When we look at that vignette, we see that these two paid people have reached out for somebody to help them …. they're asking for expertise without acknowledgement, without any reimbursement of any sort (Rehan).

For Rehan, not only did Jane and Claire lack the language skills and cultural knowledge necessary to do their jobs properly, but when they then ask for Sunita’s help, their failure to recognise her specific skills and cultural knowledge exacerbated their original error. For many VCFSE participants this ignoring of the expertise of voluntary sector workers was also apparent in the difficulties they encountered in being financially compensated, for instance, when they spent time attending a planning meeting, or in recruiting and supporting their clients. As the Manager for a voluntary sector support service commented:

I think one of the things that always comes out for me is the reimbursement of people’s time. It doesn't always have to be financial, it could be in some other way … because then that addresses … that kind of imbalance of power and involvement in some work (Sarah).

While both sets of participants recognised the importance of the statutory sector engaging with people who have expert knowledge about South Asian communities, there were different opinions about the best way to do this. As we have described VCFSE participants tended to frame this as part of wider patterns of systemic discrimination which included a failure to understand that the needs of South Asians were not being met and an inability to value the expertise within South Asian community organisations appropriately. The solutions that they favoured were for systemic change. In contrast, when statutory sector participants referred to unconscious bias, they tended to localise this as a problem within individual practice. As a consequence, the solutions they suggested were for health care workers to learn, for example, better chairing skills:

In our executive group where we have lay members, public contributors, we have a pause during the meeting for facilitated conversation from those people to reflect on how they are experiencing the meeting and there is no right of redress … this is very powerful and has changed the culture of that group (Kayleigh).

### “Sometimes you feel like you’re repeating yourself”: Speaking out and being ignored

While VCFSE participants in particular were aware of the shortfall in dementia services for South Asians, participants in both sets of focus groups and interviews described difficulties in drawing public attention to these inequalities. As Babar described, for many VCFSE participants their reluctance came from a concern that to do so would risk being drawn into an argument: “*It can be quite difficult, actually it is a challenge … I think they find it quite confrontational when we do ask the expressions and we do bang the drum*”. For some VCFSE participants, the reluctance of decision makers to address the accessibility of dementia services placed them in a dilemma: if they consistently raised these issues, then they risked being seen as a troublemaker:

I think it's a bit sad that Sunita didn't feel confident enough because I have been there in her place, again many times where you're the room full of white people and you are the only one that either brings up an equality issue or an inclusive issue. And you can kind of like feel, well, actually, if it wasn't for me to say something, would anybody have brought that up? … This is everybody's business in terms of being inclusive so it shouldn't be down to sort of Black and Asian people to, to bring up the issue every time they’re in a meeting (Mithali).

For some VCFSE participants this dilemma was aggravated by the power imbalance that was inherent in funding meetings. As Danni, an experienced adult care manager identified planning meetings embodied “*[A] kind of power generally because actually when you're in it you might not even realize. That’s how you make people feel.”* As a consequence, focus group participants described how Sunita needed to be empowered to raise the needs of her South Asian clients: “*if she's trying to challenge him, she needs to feel comfortable to do that, but also that she's not going to challenge if she doesn't think that they're going to listen*”. Health and social care practitioners could also be uneasy at broaching issues around equality, but for them this stemmed from a fear that they might do so in an inappropriate way:

I think that people perhaps are not confident about having that type of conversation. … [they] get concerned about saying the wrong thing or asking the questions in our sort of ham-fisted way, concerned about their own embarrassment (Lydia).

## Theme 3: Prioritising relationships

Both VCFSE and statutory sector participants saw trust as being central to partnership working at both an organisational and a personal level. In turn, developing trust required a sustained commitment on both sides over a period of time and a shared sense of values. Thus, Nida commented: “*Commissioners*, *councillors, I think sometimes they just work for themselves, not for the community, and they are actually [living] there … If you got the passion to support people regardless of culture, you go [the] extra mile*”.

### “It’s a culture change”: Organisational partnerships

Participants in both groups pointed to a wider context of partnership working beyond the development of personal relationships in which organisations agreed a broader remit for this collaboration. Within a genuine partnership, roles needed to be respectful and equally balanced so that, for instance, clear lines of communication and information sharing could be agreed. Without this then “*there can be a lack of feeling joined up together with essentially with the same ultimate goals*” (Lydia). Agreeing the outlines of partnership working at an organisational level also reduced the possibility of conflict or tensions:

If you've got an agreement before you start some work, it's kind of better because then everyone knows the boundaries and you know how far you can go and what can and can't be shared (Sarah).

However, other participants were sceptical whether workload and other pressures within the statutory services meant that it would be difficult for these services to provide the necessary commitment to partnership working:

I think services need to change and rather than say ‘come to us’ [say] ‘we need to go to them and we need to take it into the community … we need to link in with them and we need to really support them’ … So it’s a culture change, it’s the services that need to change their culture completely. To be honest with you and frank with you, whether that will happen or not I don’t know (Imad).

### “It’s a bit like marriage”: Working personal relationships

If partnerships needed to be organised at an operational level to provide a working context, then they also needed to work at a personal level. VCFSE participants described a number of potential obstacles to this building a relationship – including negotiating their way through complex networks of service commissioning and delivery to identify who to talk to:

Voluntary sectors cannot go to a mainstream organisation - I mean they don't even know who to contact most of the time (Fakhar).

Perhaps more fundamentally even when a VCFSE participant could work with the statutory sector, so the residue of previous negative experiences of working with organisations meant the development of trust took longer:

BAME communities have been disadvantaged and ignored for such a long time that they don't trust people. Now there's a trust issue. You have to build that trust. And how do you build up trust? You need to be consistent. You need to (keep) the promises you make, you need to see them through, and that's kind of what I've learnt (Imad).

For their part, some statutory sector participants were aware that their colleagues within VCFSEs might be cautious about developing relationships with the statutory sector. For Lydia it was about prioritising partnership working and taking time “*to develop relationships with different communities … to have regular conversations*” but “*it is important work, and it is worth the time to do that”*. For some statutory sector participants developing effective working partnerships also required personal skills as this dialogue illustrates:

Nat: [it’s important] to stop and think before doing it, not just to rush in there, just to spend time at the beginning as well to think about the issues.

Mitchell: So basically, it's a bit like marriage.

Nat: … So what would you want to learn from the marriage? Flexibility? Working in partnership, [be] patient.

Mitchell: Active listening, reflection.

Nat: Yes, cooperate.

Kate: Not just do as you’re told.

## Discussion

In this study we set out to identify the constraints that impact on partnership working to prevent South Asian led community groups and the statutory services from working together effectively. We generated three vignettes about commonly experienced challenges to partnership working and presented these to a series of focus groups and interviews with 13 VCFSE and 16 participants working in the statutory sector. Using deductive thematic analysis, we grouped responses into three themes: White British-centric services, unconscious bias or structural racism and prioritising relationships.

According to Boydell and Rugkåsa (2007) successful partnership working occurs when partners connect with one another, develop a more holistic understanding of the priorities of local communities and build trust in each others’ expertise. Working together on a common problem enables both sides of the partnership to do their jobs better building more effective projects and improved service delivery. Our findings are consistent with a series of studies which point to the White British-centric nature of dementia services (e.g., Ahmed et al., 2024; Blakey et al., 2016; Jutlla & Arblaster, 2023; Nazir and Kevern, 2024; Parveen et al., 2017) – service deficits which often propel services towards partnership working. In addition, our findings have highlighted a series of constraints that impact on joint working to limit its effectiveness.

First, while participants from statutory and VCFSE sectors agreed about many of the challenges in service delivery, they also had very different perspectives on what caused these problems, and therefore what solutions were required. Thus, both groups of participants recognised that statutory services often lacked many of the skills necessary to deliver person-centred care (for instance not speaking their clients’ preferred language) with some VCFSE participants also describing a lack of appropriate knowledge or empathy. Both statutory sector (e.g., Mitchell) and VCFSE (e.g., Jasprit) participants also shared a certain cynicism about the motivations of managers to create visibly inclusive working practices. Similarly, both groups of participants pointed to how a lack of time to build relationships and competing priorities could undermine the development of trust that was necessary to build effective working relationships.

Despite these commonalities the two groups of participants diverged in how they understood the meaning and significance of these service deficiencies. Statutory sector participants did not recognise the challenges outlined in the vignettes as typical (Nat) or minimised their impact (Anya) or attributed them to poor individual practice (Kayleigh). In contrast, for many VCFSE participants, the challenges that our fictional character of Sunita faced were typical of wider, systemic social inequalities that they encountered not only as professionals but also in other areas of their lives. For VCFSE participants these unconscious biases were evident in a lack of knowledge about South Asian culture and language (Nida), a failure to appropriately reimburse the expertise and time commitment of VCFSEs (Sarah, Rehan) and in stereotypical assumptions about families (Rehan). As a consequence, some VCFSE participants described being ignored (Imad) or not being heard (Nida, Mithali). As such, for VCFSE participants repeated instances of indifference and inappropriate practice pointed at best to an unconscious bias, and at worst to a structural racism within dementia services.

This divergence of opinion as to the importance of the challenges represented in the vignettes acts as a constraint on partnership working because it impedes the ability of partners to connect and to develop the holistic understanding of the needs of the community that they are delivering a service to. If, as Mitchell contends, partnership working is a ‘*bit like marriage*’ then the requisite active listening, reflection and co-operation that he, Nat and Kate described as being necessary may all too often be missing.

A second constraint on partnership working that we identified was that both groups of participants described a reluctance to explicitly draw attention to service deficiencies, albeit for different reasons. While statutory sector participants were concerned about “*saying the wrong thing*” (Lydia), VCFSE participants did not want to be identified as troublemakers (Babar). This acts as a constraint on partnership working because it risks preventing both sets of workers from openly addressing the differing contexts within which they work. This opens the door to potential misunderstandings and enables misconceptions to remain unchallenged. There is, for instance, a risk that workers within the statutory sector identify a set of beliefs or practices as, for instance, inherently ‘South Asian’ or even as ‘Islamic’ - a form of essentialism in which South Asian clients are framed within a stereotype in which they share common characteristics. Thus, as Rehan commented, a wish to care for one’s parents may be seen as a common trait arising from a collective South Asian culture, rather than a desire to avoid poor care practices which risk demeaning people with dementia. Such views risk feeding into a narrative that suggests poorer experiences and outcomes are the result of cultural differences or misunderstanding rather than due to structural factors including racism.

There is also a final, external, constraint acting on prevent partnership working, namely while both parties lacked power to effect change, there was at the same time a significant imbalance in the resources open to each sector: put simply statutory services are larger, more securely funded and have access to far greater resources than do their VCFSE counterparts. Thus, partnership working occurs in the context of a power disparity in which statutory services do not depend or rely on VCFSEs in the way that South Asian led community groups require support from the statutory sector. The differing perspectives on diagnosis, for instance, illustrate the impact of this difference in perspective. Thus, the targets set in England for NHS to diagnose at least two-thirds of the nominal dementia cases in their area does not currently take ethnicity into account. As a result, any shortfall in diagnosis rates amongst minority communities will have little impact so long as the overall level of diagnosis is above the 67% target. In contrast, for local South Asian led community groups, obtaining a diagnosis for a client requires them to access to local GPs and memory clinics and is facilitated by a good working relationship with these practitioners. As a consequence, there is a stronger imperative for these organisations to form effective working partnerships than there is for their counterparts in the NHS. Put bluntly, the VCFSE participants in our study needed partnership working with the NHS and council in a way that the latter simply did not need them.

In conclusion, statutory and VCFSE services are equipped with complementary skill sets that can enhance the lives of South Asians living with dementia. For instance, while staff working in the NHS provide access to assessment, diagnosis and NICE recommended treatments, those in the voluntary sector bring language skills, an understanding of religious practices and have trusted access to people living with dementia and their families. However, in order for effective partnerships to emerge, the constraints impacting on these partnerships need to be addressed. While a range of potential solutions have been outlined in the literature such as the concept of ‘*cultural humility*’ in which statutory services attempt to redress power imbalances through self-evaluation (Tervalon & Murray-Garcia, 1998), it is beyond the scope of this paper to address these in depth. However, elsewhere we have outlined a range of potential strategies at the level of individual workers, team managers and commissioners in a website that we developed during this study (https://raceequalityfoundation.org.uk/adapt/).

Partnership working between statutory and VCFSE organisations remains a keyway of reducing the inequalities in dementia care which research has identified time and again. On their own, neither sector has the resources or expertise required to deliver care appropriately and sustainably at any point within the dementia care pathway. Only by statutory and voluntary services coming together and overcoming the constraints that they face will it be possible to address these systemic biases in service delivery and for people from South Asian communities to have the care to which they are entitled.

## Supplemental Material

Supplemental Material - “You just can’t do that in dementia care” - Barriers to partnership working within dementia services for people from south Asian communities Supplemental Material for “You just can’t do that in dementia care” - Barriers to partnership working within dementia services for people from south Asian communities by Cheston R, Dodd E, Smith P, Woodstoke NS, Jutlla K, Fry G, Truswell D, Butt J and Parveen S in Dementia: the international journal of social research and practice

Supplemental Material - “You just can’t do that in dementia care” - Barriers to partnership working within dementia services for people from south Asian communities Supplemental Material for “You just can’t do that in dementia care” - Barriers to partnership working within dementia services for people from south Asian communities by Cheston R, Dodd E, Smith P, Woodstoke NS, Jutlla K, Fry G, Truswell D, Butt J and Parveen S in Dementia: the international journal of social research and practice

## Notes

1 https://www.ethnicity-facts-figures.service.gov.uk/uk-population-by-ethnicity/national-and-regional-populations/population-of-england-and-wales/latest#by-ethnicity.

2. Protocol number ISRCTN16939033, registered on the 19^th^ of May 2021.

3. We received ethics approval from HRA and Health and Care Research Wales on the eighth of February (IRAS project ID 289226, HRA reference: 20/HRA/6109), with minor amendments subsequently being approved on the third of November 2021 and the 10^th^ of November 2021.
