# Supplementary material for: “You just can’t do that in dementia care”: Barriers to partnership working within dementia services for people from south Asian communities
Source: Dementia (London). 2024 Sep 14;24(4):611–30. doi: 10.1177/14713012241283189 (PMC11997284; doi:10.1177/14713012241283189)
Supplement: Supplemental Material - “You just can’t do that in dementia care” - Barriers to partnership working within dementia services for people from south Asian communities [file sj-pdf-1-dem-10.1177_14713012241283189.pdf]

Appendix One – Vignettes used within focus groups.

Vignette One: acknowledging service inequality in commissioning.

Sunita manages a small community organisation providing support to older people from south Asian communities, including people who may be living with dementia. Sunita attends a planning meeting at the local council, where Anna (who works for a large, national dementia charity) makes a presentation about the network of Dementia cafés that she runs. The purpose of the meeting is to decide on funding priorities for the next financial year. The cafés are one of the main ways for families to find support after a diagnosis.

During the presentation Anna does not refer to the ethnicity of Dementia café service users, and nobody in the room asks about this. Sunita suspects that most, if not all, of the people who attend will be white British as none of the people she works with attend the Dementia cafés; they see them as being aimed solely at people from the white community.

Sunita is in two minds about whether to ask a question: she feels it is important to raise this issue as her service users would greatly benefit from improved support. However, she is concerned that as she is the only non-white person at the meeting, then the other members of the group might feel the question is politically motivated and might dismiss her concerns and view her as those of a troublemaker.

After the meeting has finished, Sunita approaches Anna and asks if the cafes will be providing any particular support to people from south Asian communities. Anna explains that now that the meeting has agreed with her plans it will be difficult to change them.

Discussion questions.

- Could anything have been done either before or during the meeting to ensure that
  - Anna’s presentation addressed the importance of equality of access?
  - That Sunita felt able to share her concerns that differences between people living with dementia from different communities were being ignored?
- What else could have occurred during the meeting to enable inequalities within service provision to be addressed more openly?
- What would be your top tip to improve the situation?

Vignette Two: acknowledging expertise in joint working.

Sunita works for a community organisation supporting older people from south Asian communities, some of whom may be living with dementia. Sunita has been helping Jane (a nurse) and Claire (a psychologist) from the local NHS Trust to adapt a short course they've developed for a group of South Asian family carers.

Sunita spent a lot of time helping Jane and Claire to adapt the course. She identified families who could benefit from the course and translated the course materials. As many families were reluctant to talk openly in a group setting, thinking it would make things worse, Sunita encouraged families to attend and was in regular contact with them between the weekly sessions to provide reassurance and to answer questions. Neither Sunita's organisation nor Sunita received any payment for this work.

At times Sunita felt that while she was always open and transparent with Jane and Claire about her work, they had not shared any information with her. When challenged Jane and Claire told her that they were not able to share what had happened during the sessions because of patient confidentiality.

Jane and Claire felt that Sunita exaggerated or sometimes made up the difficulties that South Asian carers face. They think that as many South Asian families cared for their own members, there was no need for local services to employ carers who speak Punjabi or Urdu as well.

Claire and Jane's manager was very pleased with the success of the course and wants them to continue to offer the adapted sessions. When Jane and Claire meet with Sunita to discuss repeating the course, Sunita explains that she feels that they had not truly acknowledged or recognised the time, effort and cost involved to support them with the course and is unsure whether she wants to be involved again.

Discussion questions.

- There appear to be some important differences in how Sunita, Jane and Claire approach collaboration. What solutions might help to improve the relationship between the two parties and help them to work effectively together in the future?
- Jane and Claire were surprised by Sunita's response. What do you think would help Jane and Claire understand what this was so important to her?
- Jane and Claire appear to view the programme as relevant to all carers of people living with dementia.
    - What sort of things do you think Sunita feels Jane and Claire should understand about working with her community?
    - How should Jane and Claire go about improving their understanding of how the people that Sunita works with are affected by dementia?
- How should Jane and Claire respond to Sunita?
- What would be your top tip to improve the situation?

Vignette three: sharing expertise across organisations.

Sunita works for a community organisation supporting older people from south Asian communities, some of whom may be living with dementia. Some of the service users have said that they like listening to music and singing together. Sunita looks into how other organisations have introduced music and singing sessions.

Sunita talks to Peter who coordinates and manages the local branch of a large, national dementia charity that provides singing sessions locally. Sunita and Peter have a good chat and Peter says that he will speak to his manager, Louise, about sharing their learning and experience of setting up sessions. When Peter speaks to Louise she reminds him that their main priority is to make sure he has enough volunteers available to deliver the sessions agreed with the local commissioning group. Louise points out that Peter doesn't have the capacity to support any other groups to provide what they deliver. Any new groups might also impact on their targets and future funding, and so he will have to decline these offers of collaboration.

When Sunita next speaks to Peter, he reluctantly explains that he is the only paid member of staff and his main priority is to make sure that he has enough volunteers available to deliver the sessions that have already been agreed. He says that he doesn't have the capacity to support any other groups to provide what they deliver. Instead, Peter suggests that Sunita's service users could join in the Friday group which is run at the church hall.

Discussion questions.

- What are the issues and dilemmas that Peter and Louise face in this situation?

- Why might the south Asian community group feel that Peter's offer of their clients attending the Friday sessions at the church hall would not be useful?
- What solutions might help to improve the relationship between the two parties to make it more equal? What else might Peter and Louise do to set up singing sessions for people from south Asian communities?
- What would be your top tip to improve the situation?

White board animations of these videos (not used in the focus groups) can be found at: https://raceequalityfoundation.org.uk/adapt/
