# Supplementary material for: “You just can’t do that in dementia care”: Barriers to partnership working within dementia services for people from south Asian communities
Source: Dementia (London). 2024 Sep 14;24(4):611–30. doi: 10.1177/14713012241283189 (PMC11997284; doi:10.1177/14713012241283189)
Supplement: Supplemental Material - “You just can’t do that in dementia care” - Barriers to partnership working within dementia services for people from south Asian communities [file sj-pdf-2-dem-10.1177_14713012241283189.pdf]

Appendix two: preconceived codes used for the initial analysis of workshop and interview data.

- *Shared language*, acronyms and definitions
- *Joint work* - operating together at both strategic and grassroots levels (e.g., joint delivery of services)
- *A real team* - equity of participation and accountability (not just a partnership in name only, or where the real reasons for the partnership are hidden)
- *'Professionals' Vs. 'Para-professionals'* - prejudices between different providers
- *Cultural humility* - addressing inequalities in power, avoiding tokenistic involvement of partners and enabling them to challenge statutory sector partners.
- *Development of trust* – including honesty about organisational drivers.
- *Partnership synergy* - culture of mutual organizational learning that combines the individual perspectives, resources, and skills of the partners and which recognises and values equally expertise of both partners.
- *Clear, shared and achievable objectives* in terms of outcomes that are properly resourced and changed as necessary (e.g., crisis resolution Vs. preventative support).
- *Clear identifiable leadership* that attends to practical difficulties and has good communication (e.g., management styles, governance, responsibilities and vision for the future)
- *'Pilot' Project fatigue* - resistance to embrace services which are temporary.
- *'Contractual competitiveness'*
- *Data sharing*

- *Joint ownership* of the product or outcomes of the collaborative working.
